# Blood Letting

**Published:** 1874-12

**Authors:** Rawdon Macnamara


Blood-Letting—*By Dr. Rawdon Macnamara.*—"Some time ago I had a patient under my care suffering under urgent symptoms of impending suffocation, consequent upon acute inflammation of the upper portion of the larynx and adjacent parts. A consultation was held of surgeons of great operative ability and also of great experience. All were willing to sanction my opening the trachea, but not one would sanction my opening a vein at the bend of the elbow. However, not being as thoroughly impressed as perhaps I should have been with the importance of the doctrine of the change of type in diseases, I insisted upon bleeding my patient; and never shall I forget his sense of relief as ounce after ounce escaped into the cup, until at last he exclaimed, 'Thank God, I can breathe now as well as ever I did;' and from that out his convalescence was uninterrupted.—*Lancet.*

